# The effect of introducing a financial incentive to promote application of fluoride varnish in dental practice in Scotland: a natural experiment

**DOI:** 10.1186/s13012-018-0775-0

**Published:** 2018-07-11

**Authors:** Wendy Gnich, Andrea Sherriff, Debbie Bonetti, David I. Conway, Lorna M. D. Macpherson

**Affiliations:** 1 Community Oral Health Section, School of Medicine, Dentistry and Nursing, College of Medicine, Veterinary and Life Sciences (MVLS), University of Glasgow, 378 Sauchiehall Street, Glasgow, G2 3JZ UK; 2 Dental Health Services Research Unit, Dundee Dental Education Centre, Frankland Building, Small’s Wynd, Dundee, DD1 4HN UK

**Keywords:** Evidence-based practice, Clinical guidelines, Financial incentive, Behaviour change, Natural experiment, Theoretical Domains Framework (TDF), Prevention, Fluoride varnish, Dental caries, Childsmile

## Abstract

**Background:**

Financial incentives are often used to influence professional practice, yet the factors which influence their effectiveness and their behavioural mechanisms are not fully understood. In keeping with clinical guidelines, Childsmile (Scotland’s oral health improvement programme) advocates twice yearly fluoride varnish application (FVA) for children in dental practice. To support implementation Childsmile offered dental practitioners a fee-per-item payment for varnishing 2–5-year-olds’ teeth through a pilot. In October 2011 payment was extended to all dental practitioners. This paper compares FVA pre- and post-roll-out and explores the financial incentive’s behavioural mechanisms.

**Methods:**

A natural experimental approach using a longitudinal cohort of dental practitioners (*n* = 1090) compared FVA pre- (time 1) and post- (time 2) financial incentive. Responses from practitioners who did not work in a Childsmile pilot practice when considering their 2–5-year-old patients (novel incentive group) were compared with all other responses (continuous incentive group). The Theoretical Domains Framework (TDF) was used to measure change in behavioural mechanisms associated with the incentive. Analysis of covariance was used to investigate FVA rates and associated behavioural mechanisms in the two groups.

**Results:**

At time 2, 709 74%, of eligible responders, were followed up. In general, FVA rates increased over time for both groups; however, the novel incentive group experienced a greater increase (β [95% CI] = 0.82 [0.72 to 0.92]) than the continuous incentive group. Despite this, only 33% of practitioners reported ‘always’ varnishing increased risk 2–5-year-olds’ teeth following introduction of the financial incentive, 19% for standard risk children. Domain scores at time 2 (adjusting for time 1) increased more for the novel incentive group (compared to the continuous incentive group) for five domains: knowledge, social/professional role and identity, beliefs about consequences, social influences and emotion.

**Conclusions:**

In this large, prospective, population-wide study, a financial incentive moderately increased FVA in dental practice. Novel longitudinal use of a validated theoretical framework to understand behavioural mechanisms suggested that financial incentives operate through complex inter-linked belief systems. While financial incentives are useful in narrowing the gap between clinical guidelines and FVA, multiple intervention approaches are required.

**Electronic supplementary material:**

The online version of this article (10.1186/s13012-018-0775-0) contains supplementary material, which is available to authorized users.

## Background

A substantive gap exists between what is known about and what is done about improving health [1]. Professional guidelines translate the complexity of scientific research results into recommendations that can enhance healthcare [2, 3]. Yet despite widespread agreement that guidelines are an essential foundation for effective health care delivery [2, 4], studies frequently demonstrate low compliance with guidelines [5–7]. This has led to an increasing emphasis on attempts to identify and influence the determinants of practice associated with guideline adherence [2, 7–9].

Within this context, the ability of financial incentives to influence professional practice and ultimately improve patient outcomes has been widely considered. Financial incentives may be used to increase the use of evidence-based treatments or to stimulate behaviour change in relation to preventive, diagnostic and treatment decisions [10]. However, despite ‘popular opinion’ and frequent implementation, robust evidence of the effectiveness of financial incentives as a professional behaviour change strategy is limited [10–12], and although there is evidence that financial incentives are modified through indirect belief pathways [13], the mechanisms through which they operate are not fully understood [10].

Scotland’s child oral health improvement programme (Childsmile) provided an opportunity to evaluate a financial incentive aimed at increasing compliance with clinical guidelines. Although preventable, dental caries is the most common chronic disease of childhood [14, 15] and a costly global problem which can impact on children’s health and quality of life [16–18]. While the Scottish Government’s preventive initiative (Childsmile) has led to improvement in children’s oral health, considerable inequalities remain [19] and dental extractions are the most common reason for elective hospital admissions for children [20].

This paper focuses on a key component of Childsmile—the application of fluoride varnish to children’s teeth in dental practice. There is considerable evidence that fluoride varnish application (FVA) can prevent tooth decay when used in combination with regular brushing with fluoride toothpaste [21–23]. Childsmile advocates twice yearly FVA for children 2 years and over in dental practice. This recommendation was underscored with the release of Scottish Dental Clinical Effectiveness Programme guidelines in 2010 [24].

Recognition that passive distribution of guidelines is unlikely to result in uptake of desired behaviours by professionals [25] and awareness of the dominant restorative (rather than preventive) culture within dental practice suggested the need to further encourage practitioners to apply fluoride varnish to children’s teeth. At this time, FVA was expected as part of a general capitation fee. Building evidence supporting the widely held belief that dentists in the UK (including Scotland) respond to extrinsic rewards [26–29], and as small businesses exhibit self-interest [30], led Childsmile to introduce a fee-per-item payment for practitioners who applied varnish to the teeth of 2–5-year-olds. This payment was only available to practices who signed up to Childsmile’s pilot programme which operated in West of Scotland NHS boards. From July 2006 to September 2011, practices who signed up to Childsmile’s pilot could claim £6 per application (within any 6-month period for each individual child) for varnishing their child patients’ teeth. Practices submitted claim forms to Childsmile, and payment was made, quarterly, via NHS National Services Scotland.

In October 2011, as part of Childsmile’s national roll-out, a fee-per-item payment for practitioners who applied fluoride varnish to the teeth of their 2–5-year-old patients was added into the NHS primary care dental contract. Thus, since 2011, all dental practitioners have been able to claim £6 per varnish application (within any 6-month period for each individual child) in the same way they are paid for all other ‘items of service’ by NHS National Services Scotland [31]. Practitioners are paid directly on submission of a claim form. Claims submitted by the end of the month are paid the first week of the next month. This is largely an electronic process. This change to the payment system was widely communicated to the dental profession [32, 33].

The staged roll-out of the financial incentive afforded the opportunity to compare pre- and post-incentive FVA rates and to further understand the mechanisms through which financial incentives operate through a natural experiment. In reporting this natural experiment, this paper addresses two key questions. First, to what extent did frequency of FVA differ pre- and post-introduction of the financial incentive, and secondly, what are the underlying mechanisms through which the financial incentive may have influenced FVA rates?

It was hypothesised a priori that a greater change (from pre- to post-financial incentive) in frequency of FVA would be observed for practitioners for whom the ability to claim a fee-per-item payment was made available for the first time during the study period (those who had not worked in a Childsmile pilot practice) when considering delivery to 2–5-year-old patients (the age group for whom payment was introduced).

## Methods

### Design and participants

A natural experimental approach using a population-based longitudinal cohort of dental practitioners was undertaken in dental practice in Scotland before (time 1) and after (time 2) the roll-out of a national financial incentive aimed at promoting FVA through changes to the NHS primary care dental contract. All active dental practitioners defined as those who had submitted payment claims for dental services to NHS National Services Scotland in the 6 months prior to the roll-out were eligible to participate at time 1 (*N* = 2526). At time 2, all those who participated at time 1 (*n* = 1090) were asked to participate for a second time.

### Measures

Demographics, behavioural outcome (FVA) and potential mechanisms of behaviour change were measured at both time points via postal questionnaire. The questionnaire was piloted by a convenience sample of eight dental practitioners to ensure clarity of wording and appropriate structure and length. A few typographical errors were corrected and minor changes made to the format. The time 1 questionnaire is available (Additional file 1). Measures and format did not change at time 2.

#### Demographics

Standard protocol was used to measure practitioners’ professional status (principal dentist, associate dentist, salaried dentist, locum assistant or a vocational trainee), length of time practicing as a dentist (in years) and whether they had worked in a Childsmile pilot practice (yes/no) [34].

Three additional measures were obtained from the Management Information Dental Accounting System Database, NHS National Services Scotland, Information Services Division, for all responding dental practitioners: sex, age (in years) at time 1 and Scottish Index of Multiple Deprivation or SIMD (a national composite area-based indicator of socioeconomic status) of practice location (quintile 1 most deprived to 5 least deprived) at time 1 [35].

#### Outcome measure (self-reported frequency of FVA)

The self-reported frequency of fluoride varnish application to child patients (≤ 17 years) in dental practice was measured using a 5-point Likert-type scale (never, at very few appointments, at some appointments, at most appointments, at every appointment), for clinically relevant age, groups 2–5 years, 6–12 years and 13–17 years, respectively, and for two categories of caries risk status (standard and increased risk). Dental practitioners are routinely responsible for assessing the caries risk status (either standard or increased risk) of all their child patients via a full medical, dental and social history as outlined in professional guidelines [24, 36]. Caries prevention should then be delivered accordingly [24].

#### Potential mechanisms of change

##### Approach to measurement

The 12-domain Theoretical Domains Framework (TDF) [37] was used to measure the behaviour change mechanisms potentially operating as a result of the introduction of the financial incentive. The TDF was selected as a method of comprehensively identifying psychological and organisational factors that may influence the implementation of professional, in this case dental practitioners’, behaviour [37–41].

##### Selection of theoretical domains

In order to produce a focussed, concise questionnaire and in keeping with standard practice when using the TDF (e.g. [42–45]), a consensus group determined which domains were relevant to the behaviour being studied. A description of the 12 domains and their constructs is available [37]. The consensus group comprised health psychologists and other behavioural scientists, clinicians, dental public health specialists, health services researchers, Childsmile Executive members, policy makers and those involved in the development of clinical oral health guidelines, all with expertise in the implementation of evidence-based dental behaviour.

The consensus group identified nine domains relevant to FVA: knowledge, skills, social/professional role and identity, beliefs about consequences, motivation and goals (intention), environmental context and resources, social influences (norms), emotion and behavioural regulation. Three domains were not assessed. The domain nature of the behaviour was excluded a priori as it relates more to an understanding of the behaviour itself than to influences on it. Beliefs about capabilities was excluded as the consensus groups’ view was that dental practitioners have the knowledge and skills necessary to apply fluoride varnish. Finally, the domain, memory, attention and decision processes was excluded. Since implementation of guidelines pertaining to FVA in dental practice stipulates that varnish should be applied to the teeth of all children twice a year (and most children will only attend practice twice a year), compliance with those guidelines was viewed by the consensus group as leading to automatic delivery (with a simple check of contraindications) at all routine appointments.

##### Assessment of the theoretical domains

Constructs within agreed domains [37] were considered by the consensus group and individual questionnaire items developed to measure those constructs of relevance to the target group, behaviour and setting. As per standard practice (e.g. [42, 45, 46]), established items measuring the agreed constructs were drawn from existing literature [37] then tailored to the target group, behaviour and setting.

The consensus group reviewed items to ensure clarity and item fit within domains. By design, the number of items per domain reflected the likely influence of that domain as viewed by the consensus group. The more influential the domain was perceived to be, the more questionnaire items it had associated with it. Resultant items were both positively and negatively phrased and measured on a 7-point Likert scale. Negative items were reverse scored. The items comprising each domain, and for multi-item scales an estimate of their internal consistency (Cronbach’s alpha, calculated at time 1), are presented in Additional file 2.

##### Financial items

Two items specifically related to the financial incentive were included. Respondents were asked (on a 7-point Likert-type scale) to what extent they agreed or disagreed with the following: In general, applying fluoride varnish to my child patients twice a year (as supported by current Scottish Dental Clinical Effectiveness Programme guidelines [24]) (1) ‘is something I receive appropriate compensation to do’ and (2) ‘would increase in my practice if it was more financially rewarding’, while considering patient age group (2–5, 6–12 and 13–17 years). Both financial items were included in the domain ‘beliefs about consequences’.

### Survey administration

NHS Scotland’s National Services Scotland, Information Services Division, provided a list of eligible dental practitioners and their contact details. Practitioners undertaking only orthodontic or emergency work, along with those whose list numbers were classified as temporary or locum, were excluded.

Time 1 questionnaires were first sent out on 2 August 2011. This was followed by two postal follow-ups for non-responders. Nearly all completed time 1 questionnaires were received by October 2011 (11 were received shortly after this date). At time 2, questionnaires were sent out on 1 February 2013 with two further postal follow-ups of non-responders. All time 2 questionnaires were received by June 2013. At both time points, practices with non-responders were contacted by phone to confirm contact details and encourage participation. Questionnaires were included in all mailings.

#### Statistical analyses

All statistical analyses were carried out in Stata IC (StataCorp, V14). A 10% random sample was double-entered to identify any systematic data entry errors. None were found. Items comprising each domain were scored positively, summed and an average ‘domain score’ calculated for each respondent.

Categorical data were summarised using percentages and numerical data using means (standard deviations/95% confidence intervals) or medians (Q1, Q3) depending on distributional assumptions.

FVA rates were reported using a Likert scale and were then converted to numerical scores (Never = 0, Few = 1, Some = 2, Most = 3, Every = 4).

Responses from practitioners who did not work in a Childsmile pilot practice when considering their 2–5-year-old patients (novel incentive group) were compared with all other responses (continuous incentive group). The continuous incentive group comprised responses from practitioners who had worked in a Childsmile pilot practice when reporting FVA for all age groups and those from practitioners who had *not* worked in a Childsmile pilot practice when considering FVA to patients 6 years and older.

### Assessing the effect of the financial incentive on FVA at time 2

An analysis of covariance model was used to test for a difference between the novel and continuous incentive groups in FVA at time 2 controlling for time 1 FVA and caries risk category.

### Assessing the mechanisms of the financial incentive

Analyses of covariance (ANCOVAs) were used to test for differences between the novel and continuous incentive groups in each of the nine theoretical domains at time 2, adjusted for time 1 and caries risk category. To account for multiple testing a *p* value of < 0.00001 was used. A similar approach was taken for the individual financial item measuring practitioners’ perceptions that they received appropriate compensation for applying fluoride varnish to their child patients’ teeth. This relationship was explored in isolation since this variable was hypothesised a priori to represent key mechanisms through which the financial incentive may operate.

Clustering of dental practitioners within practices was accounted for using the cluster command in Stata, with robust standard errors throughout. The intra-cluster correlation (ICC) for the primary analysis was calculated.

## Results

### Response rate

Of 2526 surveys sent out at time 1, 491 were subsequently identified as ineligible. One thousand ninety practitioners responded (54% of those eligible (*N* = 2035)). The methods and results of the time 1 survey have been published previously including the characteristics of responders versus non-responders [44]. At time 1, responders were younger, had qualified more recently and were more likely to be female and more likely to work in a Childsmile pilot practice.

At time 2, 129 practitioners from the time 1 cohort of 1090 dental practitioners were identified as ineligible (85 letters were undeliverable and 44 practitioners were not currently treating children). Seven hundred nine, 74%, of the time 1 responders eligible at time 2 were followed up approximately 1 year later (time 2). This represented 37% of those originally asked who were not known to be ineligible at either time 1 or time 2. Approximately half (*n* = 350) had worked in a Childsmile pilot practice. There was little difference in key characteristics of responders versus non-responders (Table 1).

**Table 1:** Characteristics of respondents at time 1 and time 2. Variables are self-reported from the time 1 study survey with the exception of sex, age and SIMD of practice which were extracted from the Management Information Dental Accounting System Database NHS National Services Scotland, Information Services Division, at the end of March 2011

	Time 1 *N* = 1090	Time 2 *N* = 709
Professional status at time 1		
Principal dentist	38.6 (421)	39.8 (282)
Associate dentist	51.7 (564)	51.1 (362)
Salaried dentist	9.3 (101)	8.8 (62)
Vocational trainee	0.2 (2)	0.1 (1)
Associate + salaried	0.1 (1)	0.1 (1)
Missing	1	1
Working/worked in a Childsmile pilot practice		
No	51.5 (557)	50.5 (350)
Yes	48.5 (525)	49.5 (343)
Missing	8	16
Length of time practicing years at time 1		
Median (Q1, Q3)	15 [6, 25]	16 [6, 25]
Min, max	1 to 46	1 to 46
Mean (SD)	16.4 (10.6)	16.4 (10.4)
Missing	17	10
Sex		
Male	52.8 (559)	50.0 (344)
Female	47.2 (500)	50.0 (344)
Missing	31	21
Age (years) at time 1		
Median (Q1, Q3)	39 (30, 49)	40 (30, 48)
Min, max	23, 67	23, 67
Mean (SD)	39.9 (10.6)	39.9 (10.3)
Missing	31	21
SIMD quintile of practice (national) at time 1		
Q1—most deprived	21.4 (226)	21.3 (146)
Q2	27.0 (285)	26.8 (184)
Q3	20.2 (214)	18.5 (127)
Q4	15.0 (159)	16.6 (114)
Q5—least deprived	16.4 (173)	16.9 (116)
Missing	33	22

### FVA pre- and post-financial incentive

**Fig. 1:**
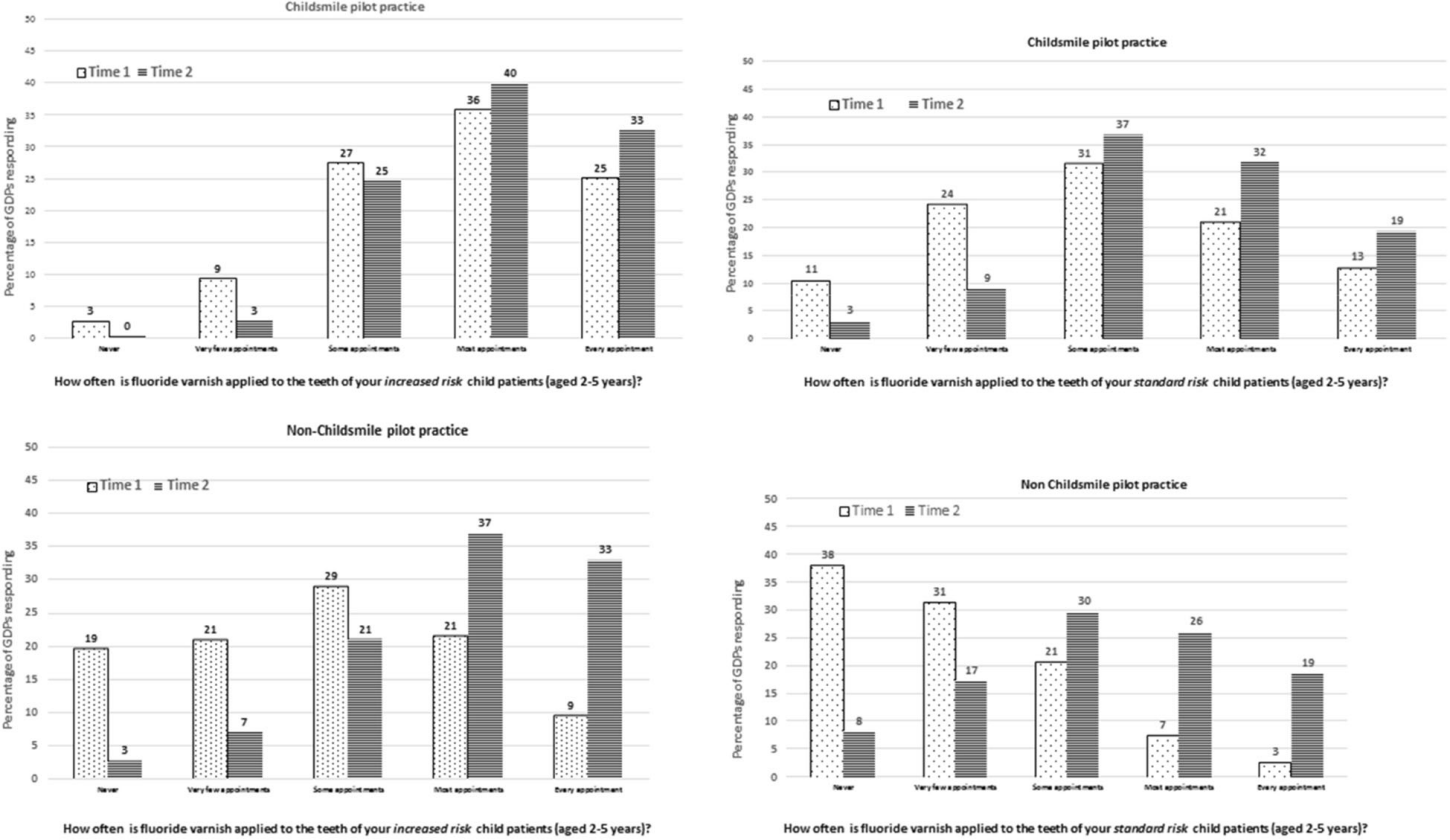
Fluoride varnish application for 2–5-year-olds by caries risk and Childsmile pilot practice status

Figure 1 presents the frequencies of self-reported FVA at time 1 and time 2 for the 709 dental practitioners who responded at both time points stratified by age group (2–5 years; 6–12 years; 13–17 years), category of risk (standard; increased) and whether the respondent had worked in a Childsmile pilot practice (yes/no). Clear differences in fluoride varnish rates emerged between groups (type of practice, patients’ age group and caries risk category) and over time.

As Fig. 1 shows, even when incentivised, self-reported FVA rates at time 2 suggest that only a third of practitioners ‘always’ apply varnish to the teeth of increased caries risk patients and less than a fifth (19%) ‘always’ apply varnish to those children assessed as having a standard risk of developing caries. However, 40% of those that had worked in a Childsmile pilot practice reported applying fluoride varnish ‘at most’ appointments for increased risk children at time 2. This figure fell to 32% for those children perceived to be at standard risk. Comparable time 2 figures for those practitioners who had not worked in a Childsmile pilot practice were 37% for increased and 26% for standard risk children. Reported varnish rates were lower for older children (Additional file 3). For example, only 1% of dental practitioners working in a Childsmile pilot practice reported applying fluoride varnish to their standard risk 13–17-year-old patients at every visit at time 1. This did not change at time 2.

After adjusting for FVA rates at time 1 and caries risk category, reported FVA rates at time 2 were higher on average for those in the novel incentive group compared to those in the continuous incentive group (β [95% CI] = 0.82 [0.72 to 0.92]). This reflected a change of almost one category on the Likert scale between the novel incentive and continuous incentive groups at time 2. The ICC was 0.34, 95% CI = [0.31 to 0.37].

### Mechanisms of change

#### Theoretical domains

The mean change in all nine domain scores between time 1 and time 2 stratified by child age group and whether the dental practitioner worked in a Childsmile pilot practice is shown in Table 2.

**Table 2:** Mean (SD) difference in Domain Scores and financial items (Time 2-Time 1) by child’s age, and Childsmile pilot status

	2–5 years				6–12 years				13–17 years			
	Non Childsmile pilot		Childsmile pilot		Non Childsmile pilot		Childsmile pilot		Non Childsmile pilot		Childsmile pilot	
	Time 1	Time 2	Time 1	Time 2	Time 1	Time 2	Time 1	Time 2	Time 1	Time 2	Time 1	Time 2
Knowledge	5.43 (1.39)	5.35 (1.57)	5.85 (1.42)	5.51 (1.50)	5.43 (1.39)	5.17 (1.53)	5.49 (1.51)	5.19 (1.47)	5.16 (1.45)	4.86 (1.54)	5.05 (1.59)	4.84 (1.52)
Mean Difference (T2-T1) [95% CI]	−0.07 [−0.22 to 0.07]		−0.34 [− 0.48 to − 0.19]		− 0.26 [− 0.40 to − 0.12]		−0.30 [− 0.45 to − 0.15]		− 0.30 [− 0.44 to − 0.15]		−0.21 [− 0.36 to − 0.06]	
Skills	4.05 (1.89)	3.99 (1.75)	3.89 (1.65)	3.97 (1.63)	5.24 (1.67)	5.36 (1.59)	5.32 (1.73)	5.52 (1.52)	5.89 (1.65)	5.96 (1.67)	6.00 (1.67)	6.21 (1.43)
Mean Difference (T2-T1) [95% CI]	−0.06 [−0.21 to 0.08]		0.08 [− 0.07 to 0.23]		0.11 [− 0.035 to 0.26]		0.20 [0.04 to 0.35]		0.07 [− 0.09 to 0.23]		0.21 [0.05 to 0.37]	
Social/professional role & identity	4.70 (1.34)	5.18 (1.28)	5.56 (1.13)	5.61 (1.56)	4.69 (1.34)	4.85 (1.27)	5.15 (1.15)	4.98 (1.29)	4.41 (1.36)	4.39 (1.34)	4.65 (1.26)	4.40 (1.44)
Mean Difference (T2-T1) [95% CI]	0.48 [0.38 to 0.59]		0.05 [−0.05 to 0.16]		0.16 [0.06 to 0.27]		−0.17 [− 0.27 to − 0.06]		−0.02 [− 0.13 to 0.09]		−0.25 [− 0.36 to − 0.14]	
Beliefs about consequences	4.92 (0.87)	5.25 (0.90)	5.39 (0.86)	5.45 (0.88)	4.98 (0.86)	5.02 (0.86)	5.24 (0.84)	5.11 (0.84)	4.76 (0.91)	4.70 (0.91)	4.88 (0.86)	4.75 (0.88)
Mean Difference (T2-T1) [95% CI]	0.33 [0.26 to 0.40]		0.06 [−0.01 to 0.12]		0.04 [− 0.03 to 0.11]		− 0.13 [− 0.20 to − 0.06]		−0.06 [− 0.13 to 0.01]		− 0.13 [− 0.20 to − 0.07]	
Environmental context & resources	4.66 (1.49)	4.92 (1.51)	4.88 (1.53)	5.10 (1.46)	4.73 (1.48)	4.92 (1.49)	4.87 (1.54)	5.04 (1.45)	4.76 (1.49)	4.94 (1.49)	4.88 (1.54)	5.06 (1.46)
Mean Difference (T2-T1) [95% CI]	0.26 [0.14 to 0.38]		0.22 [0.10 to 0.34]		0.20 [0.07 to 0.31]		0.17 [0.06 to 0.29]		0.18 [0.06 to 0.30]		0.19 [0.07 to 0.30]	
Social influences	3.94 (1.12)	4.50 (1.10)	4.63 (1.13)	4.65 (1.14)	3.98 (1.15)	4.37 (1.16)	4.49 (1.16)	4.42 (1.13)	3.93 (1.17)	4.18 (1.17)	4.28 (1.13)	4.21 (1.16)
Mean Difference (T2-T1) [95% CI]	0.57 [0.47 to 0.66]		0.02 [−0.07 to 0.12]		0.39 [0.29 to 0.49]		−0.08 [− 0.17 to 0.02]		0.26 [0.16 to 0.35]		− 0.08 [− 0.16 to 0.01]	
Emotion	4.68 (1.87)	4.99 (1.91)	5.45 (1.59)	5.41 (1.67)	4.70 (1.84)	4.73 (1.82)	5.20 (1.59)	4.94 (1.69)	4.46 (1.85)	4.31 (1.87)	4.64 (1.69)	4.45 (1.81)
Mean Difference (T2-T1) [95% CI]	0.31 [0.16 to 0.47]		−0.04 [−0.18 to 0.09]		0.03 [−0.12 to 0.17]		− 0.25 [− 0.39 to − 0.12]		−0.15 [− 0.30 to 0.01]		−0.20 [− 0.33 to − 0.06]	
Financial compensation^a^	2.46 (1.72)	3.67 (2.16)	3.24 (2.01)	4.06 (2.16)	2.42 (1.7)	2.54 (1.79)	2.70 (1.79)	3.24 (2.01)	2.41 (1.71)	2.40 (1.74)	2.63 (1.78)	2.35 (1.75)
Mean Difference (T2-T1) [95% CI]	1.21 [0.95 to 1.47]		0.83 [0.56 to 1.09]		0.12 [−0.11 to 0.35]		− 0.22 [− 0.44 to − 0.006]		−0.012 [− 0.24 to 0.21]		−0.28 [− 0.48 to − 0.07]	
Further financial reward^b^	5.12 (1.95)	4.6 (2.25)	5.24 (1.98)	4.58 (2.17)	5.06 (1.96)	4.84 (2.19)	5.28 (1.96)	5.06 (2.03)	5.02 (2.0)	4.78 (2.2)	5.24 (1.99)	5.07 (2.02)
Mean Difference (T2-T1) [95% CI]	−0.52 [−0.78 to − 0.27]		−0.66 [− 0.89 to − 0.42]		−0.22 [− 0.48 to 0.03]		0.22 [− 0.44 to 0.002]		− 0.24 [− 0.49 to 0.02]		0.17 [− 0.40 to 0.06]	
	Standard Risk						Increased Risk					
	Non CS pilot			CS pilot			Non CS pilot			CS pilot		
	Time 1	Time 2		Time 1	Time 2		Time 1	Time 2		Time 1	Time 2	
Motivation	5.38 (1.73)	5.22 (1.71)		5.80 (1.53)	5.17 (1.72)		4.49 (1.87)	4.28 (1.76)		4.74 (1.69)	4.48 (1.64)	
Mean Difference (T2-T1) [95% CI]	−0.16 [−0.29 to −0.03]			−0.62 [− 0.75 to − 0.49]			0.21 [− 0.34 to − 0. -08]			−0.26 [− 0.38 to − 0.14]		
Behavioural regulation	4.97 (1.82)	4.72 (1.71)		5.35 (1.65)	4.74 (1.75)		4.45 (1.87)	4.25 (1.71)		4.80 (1.67)	4.40 (1.66)	
Mean Difference (T2-T1) [95% CI]	−0.25 [− 0.39 to − 0.10]			−0.60 [− 0.74 to − 0.47]			−0.20 [− 0.34 to − 0.06]			− 0.40 [− 0.53 to − 0.27]		

^a^Extent of agreement or disagreement on a 7 point Likert-type scale that ‘applying FV is something I receive appropriate financial compensation to do’ for the age group considered

^b^Extent of agreement or disagreement on a 7 point Likert-type scale that ‘FVA would increase in my practice if it was more financially rewarding’ for the age group considered

For the domains social/professional role and identity, beliefs about consequences, environmental context and resources, social influences and emotion, all mean scores increased between baseline and follow-up. With the exception of two domains (skills and behavioural regulation), there was a greater increase (or lesser decrease) in domain scores over time for those practitioners who had not worked in a Childsmile pilot practice when considering their 2–5-year-old patients.

After adjusting for baseline scores in the TDF domains and caries risk category, changes in the novel incentive group (compared to the continuous incentive group) were greater for the following domains: knowledge (β [95% CI] = 0.31 [0.25 to 0.37]; + 1/3 of a unit along the Likert scale), social/professional role and identity (β [95% CI] = 0.47 [0.41 to 0.53]; + 1/2), beliefs about consequences (β [95% CI] = 0.35 [0.31 to 0.39]; + 1/3), social influences (β [95% CI] = 0.25 [0.21 to 0.29]; + 1/4) and emotion (β [95% CI] = 0.42 [0.34 to 0.49]; + 2/5) (Table 3).

**Table 3:** Results of analysis of covariance for domain scores and financial item at time 2, adjusted for time 1 and caries risk category for the novel and continuous incentive groups

TDF domain	*β*	95% CI		*p* value
Knowledge				
Time 1 covariate	0.25	0.22	0.28	
Novel incentive effect	0.31	0.25	0.37	< 0.00001
Skills				
Time 1 covariate	0.47	0.38	0.43	
Novel incentive effect	− 1.05	− 1.2	− 0.9	< 0.00001
Social/professional role and identity				
Time 1 covariate	0.64	0.60	0.67	
Novel incentive effect	0.47	0.41	0.53	< 0.00001
Beliefs about consequences				
Time 1 covariate	0.60	0.57	0.64	
Novel incentive effect	0.35	0.31	0.39	< 0.00001
Motivation and goals				
Time 1 covariate	0.35	0.32	0.38	
Novel incentive effect	0.001	− 0.07	0.07	0.954
Environmental context and resources				
Time 1 covariate	0.25	0.21	0.29	
Novel incentive effect	0.02	− 0.01	0.04	0.277
Social influences				
Time 1 covariate	0.40	0.36	0.44	
Novel incentive effect	0.25	0.21	0.29	< 0.00001
Emotion				
Time 1 covariate	0.42	0.39	0.46	
Novel incentive effect	0.42	0.34	0.49	< 0.00001
Behavioural regulation				
Time 1 covariate	0.20	0.17	0.23	
Novel incentive effect	− 0.001	− 0.07	0.07	0.984
Financial compensation ^+^				
Time 1 covariate	0.37	0.33	0.40	
Novel incentive effect	0.98	0.83	1.14	< 0.00001

^+^Extent of agreement or disagreement on a 7-point Likert-type scale that ‘applying FV is something I receive appropriate financial compensation to do’

#### Individual financial items

For the financial item *(FVA) is something I receive appropriate financial compensation to do*, all but one mean score was below 4, with most below 3, suggesting that the financial reward in place was not considered adequate compensation to a greater or lesser degree (Table 2). On average, there was a greater increase in agreement with this item over time (time 2 − time 1) when those practitioners who had not worked in a Childsmile pilot practice considered their 2–5-year-old patients. An ANCOVA modelling score on this item at time 2, adjusted for time 1 and caries risk category, confirmed that those in the novel incentive group showed greater improvement in scores compared to the continuous incentive group (β [95% CI] = 0.98 [0.83 to 1.14]; + 1 unit along the Likert scale) (Table 2).

For the financial item *(FVA) would increase in my practice if it was more financially rewarding*, mean scores ranged from 4.60 to 5.28, although not strong, suggesting some extent of agreement with this item (Table 2).

## Discussion

The aim of this large, prospective, population-wide study was to assess the effect of a financial incentive designed to increase delivery of fluoride varnish to children’s teeth in dental practice in Scotland in line with clinical guidelines. This paper considers, first, change in frequency of FVA pre- and post-introduction of the financial incentive and, second, the potential mechanisms of change through which the incentive may have influenced preventive practice. While introduction of a financial incentive was found to increase FVA rates, the low magnitude of change over time suggests a need for further intervention. Novel use of a validated theoretical framework to understand mechanisms of change suggested that financial incentives operate through complex inter-linked belief systems.

### Change in FVA (pre- to post-financial incentive)

Scottish Dental Clinical Effectiveness Programme guidelines advocate the application of fluoride varnish twice a year to the teeth of all children 2 years of age and over (irrespective of caries risk) who attend general dental practice. Yet, even when a fee-per-item payment was made available, dental practitioners’ self-reported FVA rates suggested that only a third apply varnish to the teeth of increased caries risk patients at every visit and less than a fifth to those children assessed as having a standard risk of developing caries. Reported varnish rates were even lower for older children. While this study measured frequency of FVA on an ordinal scale, it would be expected that since most children will not attend practice more than twice a year, practitioners would have to apply varnish at ‘every’ appointment if guidelines are to be met. These findings concur with recent national data showing that in 2015/2016, just 18% of 2–5-year-old children registered with a dentist received two applications of fluoride varnish per year [47].

Nonetheless, sub-group changes in FVA rates over time demonstrated a pattern commensurate with a positive effect of the financial incentive. The biggest increase in FVA was reported by practitioners who had not worked in a Childsmile pilot practice when considering 2–5-year-old patients, the age group for which a fee was introduced.

While the findings provide some support for growing evidence of the influence of remuneration on healthcare professionals, including dentists’ behaviour [10, 11, 27–29, 48], the low absolute rates of FVA and low magnitude of change over time suggest there is still a need for further intervention to ensure all children in Scotland are getting preventive treatment in line with clinical guidelines.

### Mechanisms of the financial incentive

Trends in domain scores over time generally showed a greater positive shift in beliefs which may influence FVA, when those practitioners who had not worked in a Childsmile pilot practice considered their 2–5-year-old patients, than for all other responses. This pattern of results is in keeping with a positive effect of introducing a financial incentive.

After adjusting for time 1 scores in the TDF domains, changes in the novel incentive group (compared to the continuous incentive group) were greater for five domains (knowledge, social/professional role and identity, beliefs about consequences, social influences and emotion). Beliefs related to constructs within these domains may have been influenced by the introduction of the financial incentive during the study period.

The greatest increase in domain scores for the novel (compared to continuous) incentive group was seen for change in the domain social/professional role and identity which measured practitioners’ beliefs that FVA was an important part of their own (or their team’s role). It is plausible that offering a fee-for-service payment for FVA may reinforce practitioners’ beliefs that varnish application was their responsibility. The influence of this domain in predicting dental practitioners’ preventive practice has been previously established [13, 45] and an association between perceived professional role and the effectiveness of financial rewards found [49].

Emotion had the second biggest positive difference in domain scores over time for the novel versus continuous incentive groups. This domain was measured by a single item asking practitioners to what extent applying varnish to the teeth of their child patients at least twice a year was something they really wanted to do. Again, it seems intuitive that the introduction of a specific fee for applying varnish may increase practitioner’s agreement with this statement.

Scores in the knowledge domain at time 2 (controlling for time 1) were also found to have a greater positive increase in the novel incentive group. It may be that inclusion of a specific payment for FVA has underscored that applying varnish twice a year is supported by clinical guidelines and/or that introduction of the incentive may have prompted practitioners to further educate themselves in relation to relevant guidelines. Knowledge that varnish is advocated in current guidelines has been found to be associated with varnish application rates [44].

In light of the financial nature of the intervention and studies highlighting the importance of the domain ‘beliefs about consequences’ [43, 50–52], in influencing professional behaviour, it is unsurprising that change in this domain was also greater in the novel incentive group. However, the difference between groups was not as great as for the domains social/professional role and identity and emotion. This domain measured anticipated consequences for the practitioner, their practice and their patients including perceived financial gain.

The final domain that showed a greater positive increase between time 1 and time 2 for the novel (compared to continuous) incentive group was social influences. This domain measured practitioners’ perceptions of whether their patients and colleagues wanted varnish applied. That decisions to deliver care are influenced by colleagues and patients has been previously demonstrated for a range of professional behaviours including FVA [43, 45, 50, 51]. The presence of a financial incentive may influence the extent to which FVA is viewed as desired by others. While this finding is difficult to interpret, it may be that the influence of this domain is due to increased collegiate desire of varnish application due to the financial incentive.

Individual analysis of the financial variable representing practitioners’ views of whether they received appropriate financial compensation for applying varnish to their child patient’s teeth were commensurate with a positive effect of the incentive. There was a greater positive change in practitioners’ agreement that they received appropriate compensation (at time 2 accounting for time1 scores) for the novel incentive group.

However, it is worth noting that even when the option of claiming a fee per item for applying varnish was open to them, in general, dental practitioners agreed that FVA would increase in their practice if they were paid more. This suggests that at least some practitioners did not consider the fee sufficient to compensate them. This is in keeping with a qualitative study of Scottish dental practitioners which found that the fee was insufficient to remunerate them for the time involved [13]. This qualitative study also found that the process of claiming a payment could inhibit FVA. A higher fee may have had a larger impact on the beliefs that influence decision-making and difficulty with the claim system or even perceptions of the difficulty of claiming could have mitigated against a larger intervention effect. Exploring the extent to which the monetary value of the fee and the process for claiming it influenced its effectiveness was beyond the scope of this study.

The importance of considering the appropriate amount of incentive is often raised, although few studies have compared financial incentives of different strengths [10, 11, 53]. The appropriate amount of financial reward and the likely success of the intervention will depend on the context within which the incentive is operationalised. For example, an interaction between the value placed on a behaviour and incentive strength has been proposed. It may be that a weak incentive to perform a valued behaviour is more effective than a strong incentive to perform a behaviour that is not regarded important [10]. The financial incentive within this study was operationalised within the context of an engrained restorative culture within dental practice in Scotland.

Overall, the results suggest that financial incentives impact on professional behaviour by influencing complex inter-linked belief systems. In contrast to the sole mechanism being the expectation of direct financial reward, it seems that the introduction of a monetary payment influenced practice through changes to dental practitioners’ wider perceptions relating to the context and culture in which they work. While the mechanisms through which financial incentives change behaviour have been under-theorised and investigated, the findings are in keeping with evidence that incentives operate through indirect belief pathways, for example by normalising or validating the behaviour as part of the professional’s role [13].

### Strengths and limitations

This study has several strengths, not least its scale. Its focus was a population-wide intervention, with explanatory and response  variables measured through a census of dental practitioners and their longitudinal follow-up.

The study benefitted from external validity and added to inconclusive literature on the effectiveness of financial incentives as a guideline implementation strategy. While the response rate at time 2 (74%) was very favourable when compared with contemporary surveys of dental practitioners (which achieved returns ranging from 29 to 45%) [45, 54, 55], just 37% of those originally asked (and who were not known to be ineligible) remained in the cohort. Selection bias cannot therefore be ruled out.

In addition, the national roll-out of the financial incentive precluded a randomised research design. The division of practices at time 1 to Childsmile pilot practices (where dental practitioners could claim a direct fee for varnishing their child patients’ teeth) versus all other practices (with no fee offered) provided a ‘natural’ intervention (novel incentive) and control (continuous incentive) group. However, since these groups had known and possibly unknown differences and secular trends may have influenced the results, caution in attribution and interpretation of outcomes is warranted. While the prospective cohort provides some confidence in the temporality of observed relationships, direct causality could not be inferred.

This study addressed the paucity of literature illuminating mechanisms through which financial incentives operate [10, 12], importantly through the use of a validated framework. Moreover, while the TDF has been used prospectively to facilitate implementation of health interventions and retrospectively in theory-based process evaluation [40, 56], its longitudinal use to explore behavioural mechanisms over time was novel. Given that the TDF has proven to be a comprehensive, flexible tool shown to facilitate better understanding of the pathways through which behavioural change occurs [40, 41, 56], its use was an innovative but appropriate way to further understand how financial incentives operate.

However, although this study added to the evidence that the TDF can be used across different settings and in different ways to better understand the implementation of interventions [41, 56], the interpretation of results was sometimes difficult. While a quantitative approach was chosen as it facilitated population-wide investigation, with hindsight, a mixed-methods approach incorporating a longitudinal qualitative sub-cohort would have provided further insight into the complex behavioural mechanisms underpinning the intervention.

Additionally, while a priori specification of the behavioural domains likely to act as behavioural mechanism of the financial incentive may have strengthened attributional arguments and may have been possible on the basis of consensus group expertise, a broader exploratory approach (with the exception of initial domain exclusion) was adopted due to the novel nature of utilising the TDF to explain the mechanisms of an intervention longitudinally and lack of robust research evidence specific to the study setting on which to inform hypothesis.

The consensual nature of the TDF approach also merits consideration. Initial domain selection and the selection of narrow items to assess broad domains, although informed by experts, were subjective. Moreover, although internal consistency of domains was high and expert input ensured that measures were specifically relevant to FVA, the allocation of items to domains may elicit theoretical debate [42].

Finally, the nature of the behavioural measure comprising the main study outcome (FVA) is worth considering. It was not possible to obtain an objective measure of FVA for all dental practitioners at the outset of this study, instead relying on self-report. However, there is no strong basis for the hypothesis that self-report bias would change between time 1 and time 2 or that there would be differential bias (or change in bias) between Childsmile pilot and non-pilot practices.

### Further research

There are calls for further robust randomised studies to assess the impact of financial incentives on professional behaviour [10, 11]. While this is key, in keeping with recent guidance on the evaluation of complex interventions [57], such studies would benefit from a critical realist perspective combining a focus on rigorous measurement of outcomes with further, qualitative or mixed methods effort, underpinned by theory, to fully understand the mechanisms through which financial incentives work. Quantitative studies using the TDF would benefit from building on, now available, validated measures [58].

Future studies should consider the strength of the financial incentive, ensure that the claim process does not mitigate against the intervention and consider the context in which the incentive is introduced. It is important to determine the minimal incentive required to effect change in order that policy makers can implement the most cost-effective strategy. Additionally, the introduction of a fee-for-service payment for FVA into the NHS primary care dental contract gives the potential to use an objective measure of FVA in the future.

Finally, further intervention is needed to ensure children in Scotland are getting preventive treatment in line with clinical guidelines. This study and others utilising the time 1 data reported in this paper have gone someway to informing the approach required [13, 44, 45], but further work is needed to develop and test theoretically informed interventions.

## Conclusion

This large population-wide study demonstrated a positive association between the introduction of a financial incentive and dental practitioners’ compliance with FVA guidelines. However, the low levels of FVA post-incentive and the small magnitude of change in FVA evidences further need to increase the frequency with which dental professionals in Scotland apply fluoride varnish. Novel longitudinal use of the TDF suggested that financial incentives operate by altering complex inter-linked belief systems. While financial incentives are likely to be a component of a successful intervention strategy, multiple approaches are required to narrow the gap between practitioners’ behaviour and current clinical guidelines.

## Additional files

Additional file 1: Survey questionnaire. (PDF 282 kb)

Additional file 2: Item composition of theoretical domain scales. (DOC 61 kb)

Additional file 3: Frequency of fluoride varnish application for children 6 years and over by caries risk and Childsmile pilot practice status at time 1 and time 2. (DOCX 92 kb)

## Abbreviations

FVA(s): Fluoride varnish application(s)
NHS: National Health Service
TDF: Theoretical Domains Framework

## References

[1] Holmes B, Scarrow G, Schellenberg M (2012). Translating evidence into practice: the role of health research funders. Implement Sci.

[2] Shekelle P, Woolf S, Grimshaw JM, Schunemann HJ, Eccles MP (2012). Developing clinical practice guidelines: reviewing, reporting, and publishing guidelines; updating guidelines; and the emerging issues of enhancing guideline implementability and accounting for comorbid conditions in guideline development. Implement Sci.

[3] Institute of Medicine (2011). Clinical practice guidelines we can trust.

[4] Francke AL, Smit MC, de Veer AJ, Mistiaen P (2008). Factors influencing the implementation of clinical guidelines for health care professionals: a systematic meta-review. BMC Med Inform Decis Mak.

[5] Gagliardi AR, Brouwers MC, Palda VA, Lemieux-Charles L, Grimshaw JM (2011). How can we improve guideline use? A conceptual framework of implementability. Implement Sci.

[6] Grimshaw J, Eccles M, Thomas R, MacLennan G, Ramsay C, Fraser C, Vale L (2006). Toward evidence-based quality improvement. Evidence (and its limitations) of the effectiveness of guideline dissemination and implementation strategies 1966–1998. J Gen Intern Med.

[7] Grol R, Wensing M (2004). What drives change? Barriers to and incentives for achieving evidence-based practice. Med J Aust.

[8] Clarkson JE, Ramsay CR, Eccles MP, Eldridge S, Grimshaw JM, Johnston M, Michie S, Treweek S, Walker A, Young L (2010). The translation research in a dental setting (TRiaDS) programme protocol. Implement Sci.

[9] Rainbird K, Sanson-Fisher R, Buchan H (2006). Identifying barriers to evidence uptake.

[10] Flodgren G, Eccles MP, Shepperd S, Scott A, Parmelli E, Beyer FR. An overview of reviews evaluating the effectiveness of financial incentives in changing healthcare professional behaviours and patient outcomes. Cochrane Database Syst Rev. 2011:CD009255. 10.1002/14651858.CD009255. 10.1002/14651858.CD009255 PMC4204491 21735443

[11] Brocklehurst P, Price J, Glenny AM, Tickle M, Birch S, Mertz E, Grytten J (2013). The effect of different methods of remuneration on the behaviour of primary care dentists. Cochrane Database Syst Rev.

[12] McDonald R (2014). Paying for performance in healthcare organisations. Int J Health Policy Manag.

[13] Ross A, Sherriff A, Kidd J, Gnich W, Deas L, Anderson, Macpherson LM: A systems analysis of fluoride varnish application in general dental practice in Scotland using the functional resonance analysis method. Appl Ergon 2018, 68: 294–303. 10.1016/j.apergo.2017.12.005 PMC5817000 29409648

[14] Petersen PE (2003). The World Oral Health report: continuous improvement of oral health in the 21st century—the approach of the WHO Global Oral Health Programme. Community Dent Oral Epidemiol.

[15] Bagramian RA, Garcia-Godoy F, Volpe AR (2009). The global increase in dental caries: a pending public health crisis. Am J Dent.

[16] Locker D, Jokovic A, Stephens M, Kenny D, Tompson B, Guyatt G (2002). Family impact of child oral and oral-facial conditions. Community Dent Oral Epidemiol.

[17] Sheiham A (2006). Dental caries affects body weight, growth and quality of life in pre-school children. Br Dent J.

[18] Nuttal NM, Steele JG, Evans D, Chadwick B, Morris AJ, Hill K (2003). The reported impact of oral condition on children in the United Kingdom. Br Dent J.

[19] Macpherson LM, Anopa Y, Conway DI, McMahon A. National supervised toothbrushing program and dental decay in Scotland. J Dent Res. 2012; 10.1177/0022034512470690 23264611

[20] Childhood admissions summary by health board of residence and speciality. Available from: http://www.isdscotland.org/Health-Topics/Hospital-Care/Inpatient-and-Day-Case-Activity/ Accessed 12 Sept 2017.

[21] Marinho VC, Higgins JP, Sheiham A, Logan S. Combinations of topical fluoride (toothpastes, mouthrinses, gels, varnishes) versus single topical fluoride for preventing dental caries in children and adolescents. Cochrane Database Syst Rev. 2004;1:CD002781. 10.1002/14651858.CD002781.pub2 PMC6999808 14973992

[22] Marinho VC (2009). Cochrane reviews of randomized trials of fluoride therapies for preventing dental caries. Eur Arch Paediatr Dent.

[23] Scottish Intercollegiate Guidelines Network (2005). Prevention and management of dental decay in the pre-school child: a national clinical guideline.

[24] Scottish Dental Clinical Effectiveness Programme (2010). Prevention and management of dental caries in children: dental clinical guidance.

[25] Grimshaw JM, Thomas RE, MacLennan G, Fraser C, Ramsay CR, Vale L, Whitty P, Eccles MP, Matowe L, Shirran L (2004). Effectiveness and efficiency of guideline dissemination and implementation strategies. Health Technol Assess.

[26] Crossley ML, Mubarik A (2002). A comparative investigation of dental and medical student’s motivation towards career choice. Br Dent J.

[27] Chalkley M, Tilley C (2006). Treatment intensity and provider remuneration: dentists in the British National Health Service. Health Econ.

[28] Chalkley M, Tilley C, Young L, Bonetti D, Clarkson J (2010). Incentives for dentists in public service: evidence from a natural experiment. J Public Adm Res Theory.

[29] Clarkson JE, Turner S, Grimshaw JM, Ramsay CR, Johnston M, Scott A, Bonetti D, Tilley CJ, Maclennan G, Ibbetson R (2008). Changing clinicians’ behavior: a randomized controlled trial of fees and education. J Dent Res.

[30] Crilly T, Le Grand JJ (2004). The motivation and behaviour of hospital trusts. Soc Sci Med.

[31] NHS General Servies Scotland. Statement of Dental Remuneration. Amendment No 135. October 2017. http://www.scottishdental.org/professional/statement-of-dental-remuneration. Accessed 31 Mar 2018.

[32] Childsmile Dental Remuneration Booklet. NHS Health Scotland 2011. http://www.childsmile.org.uk/professionals/information-for-dental-practice-staff.aspx. Accessed 31 Mar 2018.

[33] Health and Social Care Directorate CDO Letter NHS PCA(D)(2011)5 Scottish Government, Edinburgh. 2011 http://www.child-smile.org.uk/professionals/information-for-dental-practice-staff.aspx. Accessed 31 Mar 2018.

[34] Elouafkaoui P, Bonetti D, Clarkson J, Stirling D, Young L, Cassie H (2015). Is further intervention required to translate caries prevention and management recommendations into practice?. Br Dent J.

[35] http://www.gov.scot/Topics/Statistics/SIMD. Accessed 12 Sept 2017.

[36] Scottish Dental Clinical Effectiveness Programme (2012). Oral health assessment and review.

[37] Michie S, Johnston M, Abraham C, Lawton R, Parker D, Walker A, Psychological Theory G (2005). Making psychological theory useful for implementing evidence-based practice: a consensus approach. Qual Saf Health Care.

[38] French SD, Green SE, O’Connor DA, McKenzie JE, Francis JJ, Michie S, Buchbinder R, Schattner P, Spike N, Grimshaw JM (2012). Developing theory-informed behaviour change interventions to implement evidence into practice: a systematic approach using the Theoretical Domains Framework. Implement Sci.

[39] Cane J, O’Connor D, Michie S (2012). Validation of the theoretical domains framework for use in behaviour change and implementation research. Implement Sci.

[40] Francis J, O’Connor D, Curran J (2012). Theories of behaviour change synthesised into a set of theoretical groupings: introducing a thematic series on the theoretical domains framework. Implement Sci.

[41] Atkins L, Francis J, Islam R, O’Connor D, Patey A, Ivers N, Foy R, Duncan EM, Colquhoun H, Grimshaw JM (2017). A guide to using the Theoretical Domains Framework of behaviour change to investigate implementation problems. Implement Sci.

[42] Beenstock J, Sniehotta F, White M, Bell R, Milne E, Araujo-Soares V (2012). What helps and hinders midwives in engaging with pregnant women about stopping smoking? A cross-sectional survey of perceived implementation difficulties among midwives in the North East of England. Implement Sci.

[43] Islam R, Tinmouth AT, Francis JJ, Brehaut JC, Born J, Stockton C, Stanworth SJ, Eccles MP, Cuthbertson BH, Hyde C, Grimshaw JM (2012). A cross-country comparison of intensive care physicians’ beliefs about their transfusion behaviour: a qualitative study using the theoretical domains framework. Implement Sci.

[44] Gnich W, Bonetti D, Sherriff A, Sharma S, Conway DI, Macpherson LM (2015). Use of the theoretical domains framework to further understanding of what influences application of fluoride varnish to children’s teeth: a national survey of general dental practitioners in Scotland. Community Dent Oral Epidemiol.

[45] Templeton AR, Young L, Bish A, Gnich W, Cassie H, Treweek S, Bonetti D, Stirling D, Macpherson L, McCann S (2016). Patient-, organization-, and system-level barriers and facilitators to preventive oral health care: a convergent mixed-methods study in primary dental care. Implement Sci.

[46] Taylor N, Lawton R, Conner M (2013). Development and initial validation of the determinants of physical activity questionnaire. Int J Behav Nutr Phys Act.

[47] Central Evaluation and Research Team (2012). Childsmile national headline data.

[48] Bonetti D, Chalkley M, Clarkson J, Tilley C, Young L (2008). The effect of activity-based payment on dentists’ activity: evidence from a natural experiment in the UK National Health Service. SIRE Discussion Papers.

[49] Jacobsen CB, Hvitved J, Andersen LB (2014). Command and motivation: how the perception of external interventions relates to intrinsic motivation and public service motivation. Publ Adm.

[50] Bussieres AE, Patey AM, Francis JJ, Sales AE, Grimshaw JM (2012). Identifying factors likely to influence compliance with diagnostic imaging guideline recommendations for spine disorders among chiropractors in North America: a focus group study using the Theoretical Domains Framework. Implement Sci.

[51] McSherry LA, Dombrowski SU, Francis JJ, Murphy J, Martin CM, O’Leary JJ, Sharp L, Group A (2012). ‘It’s a can of worms’: understanding primary care practitioners’ behaviours in relation to HPV using the theoretical domains framework. Implement Sci.

[52] Curran JA, Grimshaw JM, Hayden JA, Campbell B (2011). Knowledge translation research: the science of moving research into policy and practice. J Contin Educ Heal Prof.

[53] Bonetti DL (2014). Evidence not practised: the underutilisation of preventive fissure sealants. Br Dent J.

[54] Bonetti D, Pitts NB, Eccles M, Grimshaw J, Johnston M, Steen N, Glidewell L, Thomas R, Maclennan G, Clarkson JE, Walker A (2006). Applying psychological theory to evidence-based clinical practice: identifying factors predictive of taking intra-oral radiographs. Soc Sci Med.

[55] Bonetti D, Johnston M, Clarkson JE, Grimshaw J, Pitts NB, Eccles M, Steen N, Thomas R, Maclennan G, Glidewell L, Walker A (2010). Applying psychological theories to evidence-based clinical practice: identifying factors predictive of placing preventive fissure sealants. Implement Sci.

[56] Phillips CJ, Marshall AP, Chaves NJ, Jankelowitz SK, Lin IB, Loy CT, Rees G, Sakzewski L, Thomas S, To TP (2015). Experiences of using the Theoretical Domains Framework across diverse clinical environments: a qualitative study. J Multidiscip Healthc.

[57] Moore GF, Audrey S, Barker M, Bond L, Bonell C, Hardeman W, Moore L, O’Cathain A, Tinati T, Wight D, Baird J (2015). Process evaluation of complex interventions: Medical Research Council guidance. BMJ.

[58] Huijg JM, Gebhardt WA, Crone MR, Dusseldorp E, Presseau J (2014). Discriminant content validity of a theoretical domains framework questionnaire for use in implementation research. Implement Sci.

