# Supplementary material for: The effect of introducing a financial incentive to promote application of fluoride varnish in dental practice in Scotland: a natural experiment
Source: Implement Sci. 2018 Jul 11;13:95. doi: 10.1186/s13012-018-0775-0 (PMC6042272; doi:10.1186/s13012-018-0775-0)
Supplement: Supplementary file 1 — Survey questionnaire. (PDF 282 kb) [file 13012_2018_775_MOESM1_ESM.pdf]

# Application of Fluoride Varnish to Children's Teeth:

# A Follow-up Survey of Dental Practitioners in Scotland

# 2013

| |
|---|
| *Unique Respondent Identifier to be printed here* |

*You will not be identified, this unique identifier will be used to track responses and send out further mailings*

## SECTION 1: YOU AND YOUR PRACTICE

1. How would you describe yourself? *(Tick one box)*

   Principal dentist ☐ Associate dentist ☐ Salaried dentist ☐ Locum assistant ☐ Vocational trainee ☐

2. How long have you been practicing as a dentist? *(Write in numbers of years)* ☐

3. In which setting(s) is the practice you work most frequently? *(Tick all that apply)*

   General Dental Service ☐ Community Dental Service ☐ Salaried General Dental Service ☐

   Other (Please give details) ☐

4. If you work in **another** practice(s) in which other setting(s) do you work? *(Tick all that apply)*

   General Dental Service ☐ Community Dental Service ☐
   Salaried General Dental Service ☐ I work in one practice only ☐

   Other (Please give details) ☐

5. Do you currently have any patients aged 17 years or under (at any location)?

   Yes ☐ → *Please continue to Question 7.*

   No ☐ → *You have completed all relevant questions. Please turn to the instructions for returning the questionnaire on the back page.*

6. **Approximately** what % of **your** patients (at all locations) are in the following age groups? *(Write % in each box)*

   2 - 5 years of age ☐ 6 - 12 years of age ☐ 13 - 17 years of age ☐

7. Is the practice you work at **most** frequently currently, or has it ever been, a Childsmile practice? *(Tick one box)*

   No, never ☐ Yes, currently a CS Practice ☐ Yes, but <u>not</u> currently a CS Practice ☐

8. If you answered 'yes' to question 7, in what year did the practice you work at **most** become a 'CS Practice'? *(Write in year)* ☐

9. If you work in **another** practice(s), are they or have they ever been a Childsmile practice? *(Tick one box)*

   No, never ☐ Yes, currently a CS Practice ☐ Yes, but <u>not</u> currently a CS Practice ☐

   I work in one practice only ☐

## SECTION 2: CURRENT PRACTICE

**All children are <u>at risk</u> of developing dental caries, some are at <u>increased risk</u>. An assessment of <u>increased risk</u> is based on previous caries experience, residency in an area of deprivation, healthcare workers opinion and/or your knowledge of the child and history taken (including caries incidence in siblings, toothbrushing and dietary habits). Children who *<u>do not</u>* <u>have increased risk</u> are defined as '<u>standard risk</u>'.**

***Thinking of <u>all</u> your child patients (in all locations if you work in more than one)***

**<u>1. Children with increased risk of caries</u>:**

a) How often is fluoride varnish applied to the teeth of **your** child patients with **increased** risk of caries: (*Please tick one box for each age-group. Not applicable should **only** be ticked if you do not have **<u>any</u>** child patients within this age group with **increased** risk of caries).*

| | At every appointment | At most appointments | At some appointments | At very few appointments | Never | Not Applicable |
|---|---|---|---|---|---|---|
| i) 2-5 years | | | | | | |
| ii) 6-12 years | | | | | | |
| iii) 13-17 years | | | | | | |

b) In the last year, approximately what percentage of **your** child patients with **increased** risk of caries received fluoride varnish at least twice? *(Write in percentage. Please estimate as accurately as you can)*

%

c) For **my** child patients with **increased** risk of caries: *(Please circle one number for each row)*

| | **Strongly Disagree……………………………. Strongly Agree** |
|---|---|
| i) **In the next year,** I intend to increase the number who receive fluoride varnish at least twice**.** | **1 2 3 4 5 6 7** |
| ii) I already know how I can increase the number who receive fluoride varnish at least twice **in the next year.** | **1 2 3 4 5 6 7** |

**_2. Children with standard risk of caries:_**

a) How often is fluoride varnish applied to the teeth of **your** child patients with **standard** risk of caries: (*Please tick one box for each age-group. Not applicable should **only** be ticked if you do not have **<u>any</u>** child patients within this age group with **standard** risk of caries).*

| | At every appointment | At most appointments | At some appointments | At very few appointments | Never | Not Applicable |
|---|---|---|---|---|---|---|
| i) 2-5 years | | | | | | |
| ii) 6-12 years | | | | | | |
| iii) 13- 17 years | | | | | | |

b) In the last year, approximately what percentage of **your** child patients with **standard** risk of caries received fluoride varnish at least twice? *(Write in percentage. Please estimate as accurately as you can)*

%

c) For **my** child patients with **standard** risk of caries: *(Please circle one number for each row)*

| | **Strongly Disagree……………………………Strongly Agree** |
|---|---|
| i) **In the next year,** I intend to increase the number who receive fluoride varnish at least twice**.** | **1 2 3 4 5 6 7** |
| ii) I already know how I can increase the number who receive fluoride varnish at least twice **in the next year.** | **1 2 3 4 5 6 7** |

3. How often do the following dental team members apply fluoride varnish to **your** child patients: (*Please tick one box for each professional group. Only tick the last column if this professional group is not employed at **any** location in which you work)*

| | Always | Mostly | Sometimes | Rarely | Never | Not employed by the practice(s) I work in |
|---|---|---|---|---|---|---|
| a) You | | | | | | |
| b) Other GDPs | | | | | | |
| c) Dental Hygienists | | | | | | |
| d) Dental Hygienist Therapists | | | | | | |
| e) Childsmile Trained Extended Duty Dental Nurses | | | | | | |
| f) Locum Assistants | | | | | | |
| g) Vocational trainees | | | | | | |

4. What influences which dental team member applies fluoride varnish to your child patients? *(Please circle one number for each row)*

| | **Strongly Disagree.................................. Strongly Agree** | | | | | | |
|---|---|---|---|---|---|---|---|
| a) Age of patient | **1** | **2** | **3** | **4** | **5** | **6** | **7** |
| b) How cooperative the patient is | **1** | **2** | **3** | **4** | **5** | **6** | **7** |
| c) Attendance record of patient | **1** | **2** | **3** | **4** | **5** | **6** | **7** |
| d) Who is free at the appointment time | **1** | **2** | **3** | **4** | **5** | **6** | **7** |
| e) Who has the necessary knowledge | **1** | **2** | **3** | **4** | **5** | **6** | **7** |
| f) Other (please specify below) | | | | | | | |

**SECTION 3: YOUR VIEWS**

1. What are your views about applying fluoride varnish *(please provide an answer to all 4 items (a to d) for each age group)*

| | | | | | | | | | |
|---|---|---|---|---|---|---|---|---|---|
| Applying FV to **2 to 5 yr olds** is | a) Important | 1 | 2 | 3 | 4 | 5 | 6 | 7 | Unimportant |
| | b) Necessary | 1 | 2 | 3 | 4 | 5 | 6 | 7 | Not at all necessary |
| | c) Difficult | 1 | 2 | 3 | 4 | 5 | 6 | 7 | Not at all difficult |
| | d) Practical | 1 | 2 | 3 | 4 | 5 | 6 | 7 | Not at all practical |
| Applying FV to **6 to 12 yr olds** is | a) Important | 1 | 2 | 3 | 4 | 5 | 6 | 7 | Unimportant |
| | b) Necessary | 1 | 2 | 3 | 4 | 5 | 6 | 7 | Not at all necessary |
| | c) Difficult | 1 | 2 | 3 | 4 | 5 | 6 | 7 | Not at all difficult |
| | d) Practical | 1 | 2 | 3 | 4 | 5 | 6 | 7 | Not at all practical |
| Applying FV to **13 to 17 yr olds** is | a) Important | 1 | 2 | 3 | 4 | 5 | 6 | 7 | Unimportant |
| | b) Necessary | 1 | 2 | 3 | 4 | 5 | 6 | 7 | Not at all necessary |
| | c) Difficult | 1 | 2 | 3 | 4 | 5 | 6 | 7 | Not at all difficult |
| | d) Practical | 1 | 2 | 3 | 4 | 5 | 6 | 7 | Not at all practical |

2. How strongly do you agree or disagree with the following? (*Please circle one number for each age group)*

| ***In general, applying fluoride varnish to the teeth of my child patients at least twice yearly …*** | **For 2-5 year olds**<br>Strongly Disagree … Strongly Agree | **For 6-12 year olds**<br>Strongly Disagree … Strongly Agree | **For 13-17 year olds**<br>Strongly Disagree … Strongly Agree |
|---|---|---|---|
| **a) is an important part of my professional role.** | 1 2 3 4 5 6 7 | 1 2 3 4 5 6 7 | 1 2 3 4 5 6 7 |
| **b) is an important part of the role of other members of the dental team.** | 1 2 3 4 5 6 7 | 1 2 3 4 5 6 7 | 1 2 3 4 5 6 7 |
| **c) has benefits which outweigh the costs.** | 1 2 3 4 5 6 7 | 1 2 3 4 5 6 7 | 1 2 3 4 5 6 7 |
| **d) is something I really want to do.** | 1 2 3 4 5 6 7 | 1 2 3 4 5 6 7 | 1 2 3 4 5 6 7 |
| **e) will prevent caries.** | 1 2 3 4 5 6 7 | 1 2 3 4 5 6 7 | 1 2 3 4 5 6 7 |
| **f) will generally improve the oral health of a patient.** | 1 2 3 4 5 6 7 | 1 2 3 4 5 6 7 | 1 2 3 4 5 6 7 |
| **g) is not a priority for me.** | 1 2 3 4 5 6 7 | 1 2 3 4 5 6 7 | 1 2 3 4 5 6 7 |
| **h) may have undesirable consequences for the patient.** | 1 2 3 4 5 6 7 | 1 2 3 4 5 6 7 | 1 2 3 4 5 6 7 |
| **i) may have undesirable consequences for me.** | 1 2 3 4 5 6 7 | 1 2 3 4 5 6 7 | 1 2 3 4 5 6 7 |
| **j) is strongly supported by research evidence.** | 1 2 3 4 5 6 7 | 1 2 3 4 5 6 7 | 1 2 3 4 5 6 7 |
| **k) is advocated within current clinical guidelines for GDPs.** | 1 2 3 4 5 6 7 | 1 2 3 4 5 6 7 | 1 2 3 4 5 6 7 |
| **l) is something I receive appropriate financial compensation to do.** | 1 2 3 4 5 6 7 | 1 2 3 4 5 6 7 | 1 2 3 4 5 6 7 |
| **m) would increase in my practice(s) if it was more financially rewarding.** | 1 2 3 4 5 6 7 | 1 2 3 4 5 6 7 | 1 2 3 4 5 6 7 |
| **n) requires more time during a routine consultation than I have available.** | 1 2 3 4 5 6 7 | 1 2 3 4 5 6 7 | 1 2 3 4 5 6 7 |
| **o) requires more practice resources (staff/space) than I have available.** | 1 2 3 4 5 6 7 | 1 2 3 4 5 6 7 | 1 2 3 4 5 6 7 |
| **p) is not supported by my colleagues in my practice(s).** | 1 2 3 4 5 6 7 | 1 2 3 4 5 6 7 | 1 2 3 4 5 6 7 |
| **q) is difficult for my practice(s) to include in record management systems.** | 1 2 3 4 5 6 7 | 1 2 3 4 5 6 7 | 1 2 3 4 5 6 7 |
| **r) is something that is my responsibility to ensure is provided.** | 1 2 3 4 5 6 7 | 1 2 3 4 5 6 7 | 1 2 3 4 5 6 7 |
| **s) is something that children I see want.** | 1 2 3 4 5 6 7 | 1 2 3 4 5 6 7 | 1 2 3 4 5 6 7 |
| **t) is something that parents I see want for their children.** | 1 2 3 4 5 6 7 | 1 2 3 4 5 6 7 | 1 2 3 4 5 6 7 |

3. Are you aware of any guidance that relates to the application of fluoride varnish to children's teeth? (*Please write the name of the guidance below, if none please write "none")*

4. What are your main concerns in relation to applying fluoride varnish to children's teeth? (*Please specify)*

5. What are your main motivators to apply fluoride varnish to children's teeth? (*Please specify)*

6. Please add any further comments in relation to your survey responses.

**Thank you for completing this survey. Your contribution is greatly appreciated.**

Please return the questionnaire in the FREEPOST envelope provided by **1st March 2013**.

If you have any questions or require further information please contact John McHugh:

Tel: 0141 211 9853 or E-mail: john.mchugh.2@glasgow.ac.uk

## THANK YOU!
