# Supplementary material for: The effect of introducing a financial incentive to promote application of fluoride varnish in dental practice in Scotland: a natural experiment
Source: Implement Sci. 2018 Jul 11;13:95. doi: 10.1186/s13012-018-0775-0 (PMC6042272; doi:10.1186/s13012-018-0775-0)
Supplement: Supplementary file 2 — Item composition of theoretical domain scales. (DOC 61 kb) [file 13012_2018_775_MOESM2_ESM.doc]

**Additional file 2: Item composition of theoretical domain scales**

| **Theoretical Domain**  (cronbach’s alpha) | **Sub-scale Items** ^+^ |
| --- | --- |
| ***Knowledge*** | In general applying fluoride varnish to the teeth of my child patients at least twice yearly is advocated within current clinical guidelines. |
| ***Skills*** | Applying fluoride varnish is difficult (rated very difficult to not very difficult). |
| ***Social/professional role andidentity (self-standards)***  (α=0.895) | In general applying fluoride varnish to the teeth of my child patients at least twice yearly:  Is an important part of my professional role.  Is an important part of the role of other members of the dental team.  Is something that is my responsibility to ensure is provided. |
| ***Beliefs about consequences***  (α=0.920) | Applying fluoride varnish is:  Important (rated important to unimportant).  Necessary (rated necessary to not at all necessary).  Practical (rated practical to not at all practical).  In general applying fluoride varnish to the teeth of my child patients at least twice yearly:  Has benefits which outweigh costs.  Will prevent caries.  Will generally improve the oral health of a patient.  Is not a priority for me.  May have undesirable consequences for the patient.  May have undesirable consequences for me.  Is strongly supported by research evidence.  Is something I receive appropriate financial compensation to do.  Would increase in my practice if it was more financially rewarding. |
| **Motivation and goals (intention)** | For my child patient at risk of caries in the next year, I intend to increase the number who receive FV at least twice (asked for standard and high risk). |
| **Environmental context and resources (environmental constraints)**  (α=0.911) | In general applying fluoride varnish to the teeth of my child patients at least twice yearly:  Requires more time during a routine consultation than I have available.  Requires more practice resources than I have available.  Is difficult for my practice to include in record management systems. |
| **Social influences (norms)**  (α=0.862) | In general applying fluoride varnish to the teeth of my child patients at least twice yearly:  Is not supported by my colleagues in my practice.  Is something that children I see want.  Is something that parents I see want for their children. |
| **Emotion** | In general applying fluoride varnish to the teeth of my child patients at least twice yearly Is something I really want to do. |
| **Behavioural regulation** | For my child patients with risk of caries I already know how I can increase the number who receive FV at least twice in the next year (asked for standard and high risk) |
| *^+^Response options ranged from strongly disagree to strongly agree on a 7 point Likert-type rating scale and questions were asked for 2-5, 6-12 and 13-17 year olds unless stated otherwise. Negatively worded items were reverse coded.* | |
