# Supplementary figures and images for: The effect of introducing a financial incentive to promote application of fluoride varnish in dental practice in Scotland: a natural experiment
Source: Implement Sci. 2018 Jul 11;13:95. doi: 10.1186/s13012-018-0775-0 (PMC6042272; doi:10.1186/s13012-018-0775-0)

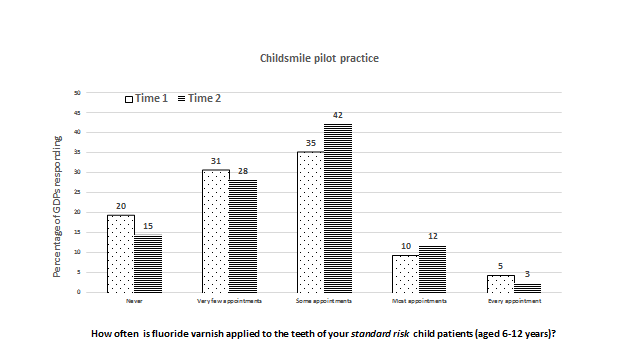


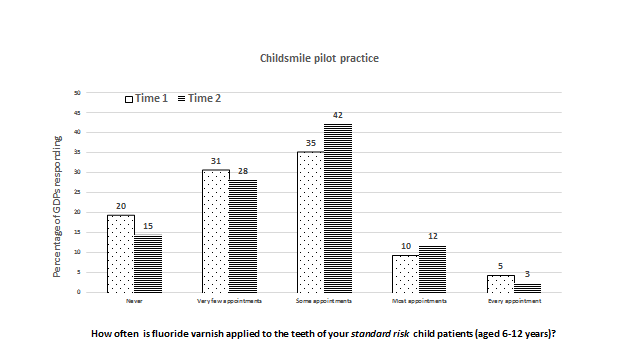


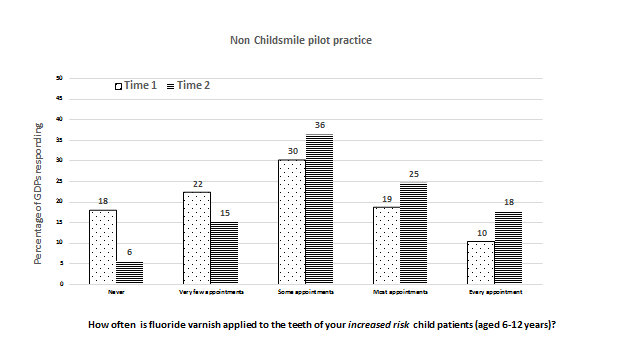


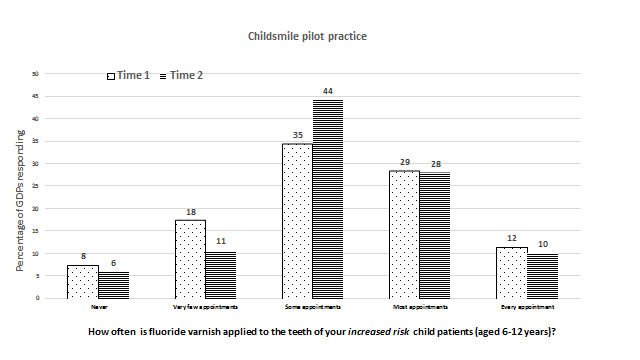


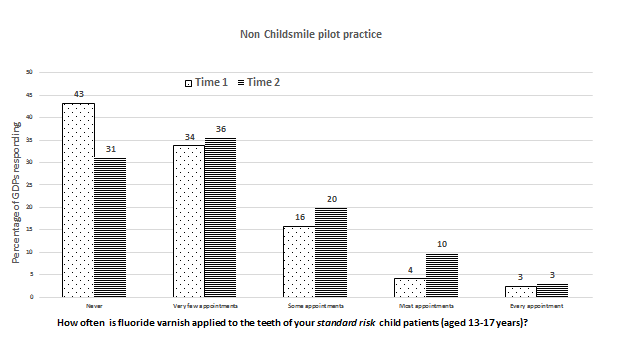


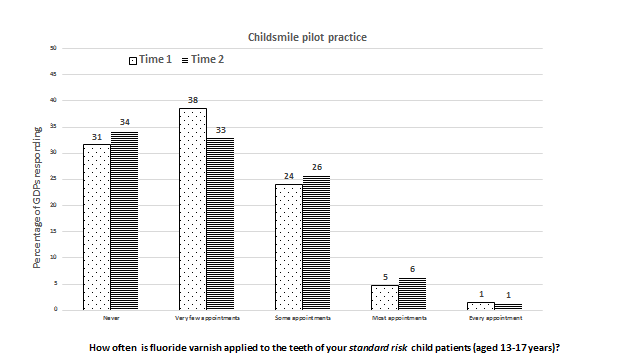


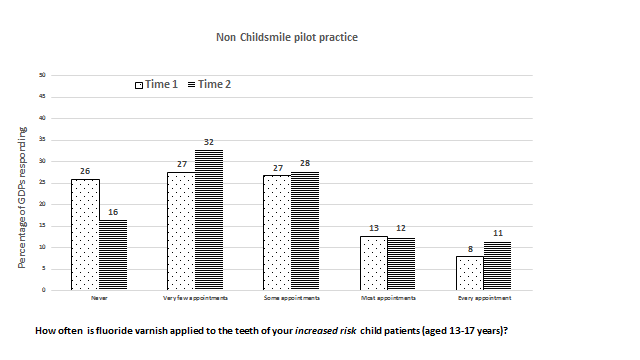


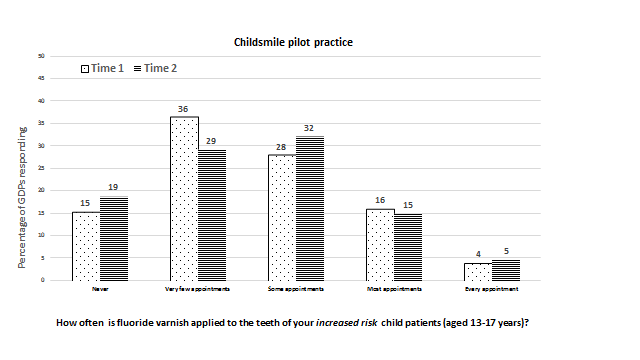

Supplement: Supplementary file 3 — Frequency of fluoride varnish application for children 6 years and over by caries risk and Childsmile pilot practice status at time 1 and time 2. (DOCX 92 kb) [file 13012_2018_775_MOESM3_ESM.docx]
